# A case of bone metastasis of colon cancer that markedly responded to S-1/CPT-11 combination chemotherapy and became curable by resection

**DOI:** 10.1186/1477-7819-4-3

**Published:** 2006-01-18

**Authors:** Kazunari Mado, Yukimoto Ishii, Takero Mazaki, Masaya Ushio, Hideki Masuda, Tadatoshi Takayama

**Affiliations:** 1 Division of Digestive Surgery, Department of Surgery, Nihon University School of Medicine, Tokyo, Japan

## Abstract

**Background:**

An oral combined fluoropyrimidine anticancer drug, tegafur/gimeracil/oteracil potassium (S-1), has recently been used alone or in combination for colon cancer.

**Case presentation:**

The patient was a 42-year-old man with sigmoid colon cancer with direct invasion of the urinary bladder and multiple costal metastases. A diagnosis of T4, M1, stage IV sigmoid colon cancer was made, and curative resection was considered impossible. S-1 at 50 mg/m^2 ^was administered by oral route from day 1 to day 14. Irinotecan (CPT-11) at 40 mg/m^2 ^was administered by intravenous day 1 and 15. This treatment was followed by 2 weeks absent period, and repeated every 4 weeks. Six cycles of administration were performed in total. Following this treatment, the multiple costal metastases resolved. Down-staging to T3, M0, stage IIA was achieved, and curative resection was judged to be possible.

**Conclusion:**

Occasional cases in which S-1/CPT-11 therapy was effective have been recently reported. The patient's tumor became resectable despite the discovery of colon cancer associated with bone metastasis at the initial examination, offering hope for cancer patients.

## Introduction

The standard chemotherapy for non-resectable advanced colon cancer is combination chemotherapy with 5-fluorouracil/leucovorin (5-FU/LV) and irinotecan (CPT-11) or with 5-FU/LV and oxaliplatin (L-OHP) [1-4]. An oral combined fluoropyrimidine anticancer drug, tegafur/gimeracil/oteracil potassium (S-1), has recently been used alone or in combination for colon cancer [5-7]. We encountered a patient with sigmoid colon cancer with multiple costal metastases, in whom S-1/CPT-11 combination therapy was effective and curative resection became applicable.

## Case presentation

The patient was a 42-year-old man with dysuria and fecaluria from late January 2004, who attended the Urology Department of our hospital. Cystoscopy and pelvic CT suggested a tumor of digestive tract origin invading the urinary bladder. The patient was referred to the Department of Digestive Surgery.

At the initial examination, height, was 160 cm; body weight, 63.5 kg; and body surface area, 1.89/m^2^. Performance status was grade 0. A fist-size tumor was palpable in the lower abdominal region. There was no particular past medical history or familial medical history.

At the initial examination, white blood cell count, was 7,600/μl; red blood cell count, 509 × 10^3^/μl; hemoglobin, 16.4 g/dl; AST, 19 IU/l; ALT, 11 IU/l; creatinin clearance, 185.9 ml/min, C reactive protein, 2.19 mg/dl; CEA, 4.3 ng/ml; and CAl9-9, 7.3 U/ml. Bacterial culture of urine detected *Escherichia coli *and *Klebsiella pneumoniae*. No malignant cells were identified on urine cytoanalysis. Pelvic computerized tomography (CT) revealed a mass lesion measuring 8 cm was present in the pelvis, with direct invasion of the posterior wall of the urinary bladder. Abdominal CT detected no space-occupying lesion in the liver or swelling of peritoneal lymph nodes.

Colonoscopy revealed a 1/2-circumferential ulcerated tumor in the sigmoid colon, and a protuberant tumor was noted on the anal side of the main tumor. Histopathologically, both tumors were well-differentiated adenocarcinoma.

**Figure 1:**
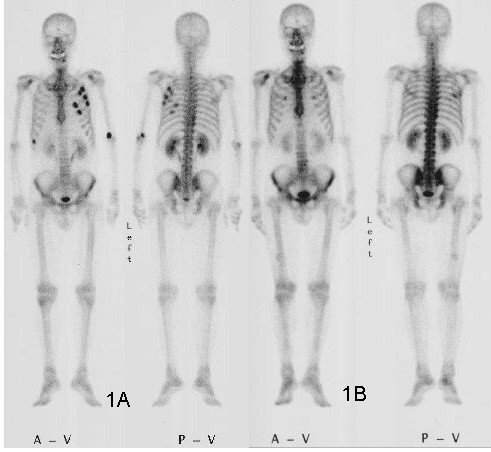
A) 99mTc-HMDP bone scintigraphy showing many lesions with accumulation in the left ribs, which were diagnosed as multiple costal metastases. B) After chemotherapy with S-1 and CPT-11, the costal metastases have resolved.

^99m^Tc-HMDP bone scintigraphy revealed many lesions with accumulation in the left ribs, which were diagnosed as multiple costal metastases (Figure 1A). Chest imaging showed no abnormal findings.

Based on the above findings, the diagnosis of T4, M1, stage IV sigmoid colon cancer was made (TNM classification), and curative resection was considered impossible.

Colostomy was performed on April 5, 2004, and chemotherapy with S-1 and CPT-11 was initiated on April 14. S-1 at 50 mg/m^2 ^was administered orally from day 1 to day 14. CPT-11 at 40 mg/m^2 ^was administered intravenously day 1 and 15. This treatment was followed by a 2 week rest, and was repeated every 4 weeks [7]. Since drug-induced liver dysfunction (grade 3) and diarrhea (grade 2) developed after completion of the 2nd cycle, the S-1 dose was reduced to 40 mg/m^2 ^after their improvement, and 6 cycles of administration were performed in total (total dose: S-1: 7,560 mg as tegafur, CPT-11: 480 mg). This therapy resulted in resolution of the multiple costal metastases (Figure 1B), and a 50% reduction of the local lesion on CT. Down-staging to T3, M0, stage IIA was achieved, and curative resection was judged to be possible. A Sigmoidectomy, lymphadenectomy, and partial cystectomy were performed on January 22, 2005.

**Figure 2:**
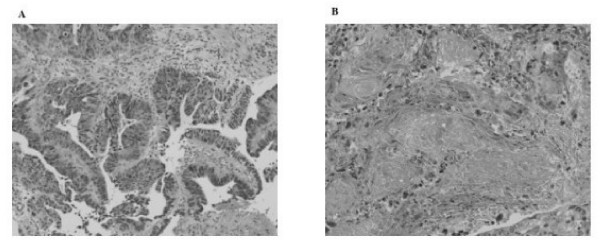
A) Microscopic image of tumor obtained by colonic biopsy before chemotherapy, showing well-differentiated adenocarcinoma. B) Microscopic image of surgical specimen after chemotherapy. Half of the tumor shows degeneration, necrosis, and fibrosis.

On histopathological examination the ulcerated tumor with a clear margin was a well-differentiated adenocarcinoma measuring 3 × 3 cm. Subserous retention of mucus was noted, but the tumor was typed as pT2, ly0, v0, pN0, indicating the possibility of curative resection. Half of the tumor was degenerative, necrotic, and fibrotic (Figure 2A and 2B). The additional cancer on the anal side was a 1.5 cm semipedunculated well-differentiated adenocarcinoma Tis. The postoperative course was uneventful, and the patient was discharged on February 4, 2005.

## Discussion

Colon cancer is often associated with metastasis to other organs such as the liver, lung, or brain. Bone metastasis, on the other hand, is relatively unusual, with a reported incidence of 4–6% [8,9] and colon cancer with bone metastasis alone without metastasis to other organs is rare.

Therapy for non-resectable advanced recurrent colon cancer has rapidly progressed since the report by Saltz *et al *[1]. The median survival time (MST) obtained with the previous 5-FU/LV therapy was about 12 months, but prolongation of MST to about 20 months by the use of 5-FU/LV, CPT-11, and oxaliplatin (L-OHP) as key drugs has been reported [2,3,10,11]. It has also been reported that the use of 5-FU/LV, CPT-11, and L-OHP together for primary or secondary therapy and thereafter has prolonged the survival time [4].

S-1 is an oral anticancer drug, which was developed based on the biochemical modulation of tegafur by gimeracil and oteracil. Gimeracil strongly inhibits dihydropyrimidine dehydrogenase, and inhibits 5-FU degradation approximately 180 times more effectively than uracil in vitro. Coadministration of gimeracil and tegafur markedly increases the antitumor activity of tegafur. Oteracil inhibits the phosphorylation of 5-FU to 5-fluorouridine-5'-monophosphate. As oteracil is distributed in the gastrointestinal tract after oral administration, it possibly decreases 5-FU-induced gastrointestinal tract toxicity. Currently, S-1 is used for cancer of the colon [5,6], stomach and lung [12][13][14,15].

Occasional cases in which S-1/CPT-11 therapy was effective have been recently reported, and a high response rate in advanced colorectal cancer has also been reported^1^. In our patient, surgery was selected after resolution of multiple bone metastases and improvement of urinary bladder invasion 7 months after the initiation of therapy. This is a rare case in which curative resection was performed despite the discovery of bone metastasis at the initial examination.

Tegafur/uracil and oral leucovorin therapy achieved survival comparable to that with 5-FU/LV therapy [16], and the usefulness of combination therapy with oral fluoropyrimidines such as Tegafiri and Tegafox has been demonstrated [17]. Treatment with monoclonal antibodies such as bevacizumab has come to be used as primary therapy [18]. The choices of chemotherapy for colon cancer may further widen in the future.

The patient's tumor became resectable despite the discovery of colon cancer accompanied by bone metastasis at the initial examination, offering hope for cancer patients. No recurrence or metastasis had occurred as of 8 months after surgery, and the patient has been receiving outpatient treatment with same regimen and is fully active.

## Competing interests

The author(s) declare that they have no competing interests.

## Authors' contributions

**KM **reviewed the literature and prepared the draft manuscript.

**YI **participated in the design of the study and helped in drafting the manuscript.

**TM**, **MU **and **HM **were involved in performing surgery.

**TT **supervised preparation of the manuscript and edited the final version for publication.

All authors read and approved the manuscript.
